# Integrated Left Ventricular Global Transcriptome and Proteome Profiling in Human End-Stage Dilated Cardiomyopathy

**DOI:** 10.1371/journal.pone.0162669

**Published:** 2016-10-06

**Authors:** Dilek Colak, Ayodele A. Alaiya, Namik Kaya, Nzioka P. Muiya, Olfat AlHarazi, Zakia Shinwari, Editha Andres, Nduna Dzimiri

**Affiliations:** 1 Biostatistics, Epidemiology and Scientific Computing Department, King Faisal Specialist Hospital and Research Centre, Riyadh, 11211, Saudi Arabia; 2 Proteomics Unit, Stem Cell Tissue Re-Engineering Program, King Faisal Specialist Hospital and Research Centre, Riyadh, 11211, Saudi Arabia; 3 Genetics Department, King Faisal Specialist Hospital and Research Centre, Riyadh, 11211, Saudi Arabia; University of California Los Angeles David Geffen School of Medicine, UNITED STATES

## Abstract

**Aims:**

The disease pathways leading to idiopathic dilated cardiomyopathy (DCM) are still elusive. The present study investigated integrated global transcriptional and translational changes in human DCM for disease biomarker discovery.

**Methods:**

We used identical myocardial tissues from five DCM hearts compared to five non-failing (NF) donor hearts for both transcriptome profiling using the ABI high-density oligonucleotide microarrays and proteome expression with One-Dimensional Nano Acquity liquid chromatography coupled with tandem mass spectrometry on the Synapt G2 system.

**Results:**

We identified 1262 differentially expressed genes (DEGs) and 269 proteins (DEPs) between DCM cases and healthy controls. Among the most significantly upregulated (>5-fold) proteins were GRK5, APOA2, IGHG3, ANXA6, HSP90AA1, and ATP5C1 (p< 0.01). On the other hand, the most significantly downregulated proteins were GSTM5, COX17, CAV1 and ANXA3. At least ten entities were concomitantly upregulated on the two analysis platforms: GOT1, ALDH4A1, PDHB, BDH1, SLC2A11, HSP90AA1, HSP90AB1, H2AFV, HSPA5 and NDUFV1. Gene ontology analyses of DEGs and DEPs revealed significant overlap with enrichment of genes/proteins related to metabolic process, biosynthetic process, cellular component organization, oxidative phosphorylation, alterations in glycolysis and ATP synthesis, Alzheimer’s disease, chemokine-mediated inflammation and cytokine signalling pathways.

**Conclusion:**

The concomitant use of transcriptome and proteome expression to evaluate global changes in DCM has led to the identification of sixteen commonly altered entities as well as novel genes, proteins and pathways whose cardiac functions have yet to be deciphered. This data should contribute towards better management of the disease.

## Introduction

Until recently, endeavors to understand alterations in disease have been directed at independent analyses of the transcriptomic or proteomic changes. These approaches preclude the notion of a direct proportional relationship between changes in transcriptomic and proteomic expression in disease, for example. While this may be correct, it has also been demonstrated that it may not always be the case, even for single cell expression under normal circumstances, as these correlations may be influenced by a number of factors, including half-lives of the entity expression or post transcriptional machinery[1–4]. Besides, in some animal disease models, bias in their correlation with clinical traits has been observed in which transcript levels appeared to correlate more strongly than protein levels [4]. Such discrepancies are likely to bear significant relevance for cardiovascular disease pathways leading to heart failure, including cardiomyopathy, cardiac hypertrophy and ischemic heart diseases, whose underlying pathways remain elusive. However, the relationship between transcript and protein levels in disease processes has not been comprehensively studied yet. On the other hand, establishing concordant proportional alterations in transcripts and proteins in a disease should be more informative and greatly enhance the deciphering of the contributing mechanisms. Specifically, despite great efforts to comprehend the underlying mechanism(s) involved in the progression of idiopathic dilated cardiomyopathy (DCM) to overt heart failure, the primary triggers for the disease remain largely undeciphered. DCM is a manifestation of the thinning of one or both ventricle(s) from an unknown cause leading to impaired cardiac contractility and therefore, overt congestive heart failure or cardiac arrhythmias. It presents an end product of myocardial damage triggered by a variety of factors such as toxic, metabolic or infectious agents, but may also be triggered by mutations in genes encoding various cardiac-related proteins[5–8]. The consequence of these manifestations is heart failure associated with a gradual increase in left ventricular end-diastolic and end-systolic volumes, wall thinning and alteration in the shape of the chambers to a more spherical and less elongated form, termed left ventricular remodeling[9]. In this process, several molecular and cellular alterations including those in the calcium (Ca^2+^) homeostasis, cyclic AMP-dependent pathways, neurohumoral activation and myofibrillar function contribute to cardiac muscle contractility and relaxation abnormalities[9–11]. Available evidence also implicates diverse pathways, such as, the vascular renin–angiotensin system, G_i_-coupled receptors, TGFβ-activin-A/Smad signaling pathway, SH2-containing cytoplasmic tyrosine phosphatase (Shp) and apoptotic signaling[10,12–14], among others, in the progression of DCM to overt heart failure. Hence, DCM is a product of interplay of changes in various interrelated signalling networks that in turn trigger multiple events in the progression to overt heart failure.

Recently, we and other laboratories have reported left ventricular transcriptome expression profiling in human DCM[15,16]. However, because of possible variabilities which may influence either transcriptome or proteome expression, the main question remains as to whether changes in these entities are congruent with each other. In order to test this notion, in the present study, we elected to investigate both global gene and protein expression in DCM from the same human left ventricular cardiac tissue harvested from identical points, employing the ABI high-density oligonucleotide microarray for the transcriptome expression profiling and the One-Dimensional Nano Acquity liquid chromatography coupled with tandem mass spectrometry on Synapt G2 system for the proteome profiling. Deciphering of such correlation patterns of alterations in transcriptional and proteomic expression should provide a valuable translational basis for elucidating some of the mechanisms involved in this vicious circle, possibly leading to the discovery of potential disease biomarkers.

## Materials and Methods

### Study patients

For the expression experiments, 300 mg of tissue were harvested from left ventricles of five DCM hearts excised from patients (3 male and 2 female; 42.3±6.3 years) with end-stage heart failure undergoing cardiac transplantation at our institution and five non-failing (NF) hearts procured from organ donors (three male and two female; 34.1±4.7 years) who died of traffic accidents with no history of cardiac disease serving as controls. All samples were procured from identical myocardial loci as described previously [15]. Fully informed written consent was obtained from all patients or family members before participating in the study, which was approved by the King Faisal Specialist Hospital and Research Centre Ethics Committee. This study was performed in accordance with the Declaration of Helsinki as adopted and promulgated by the US National Institutes of Health as well as rules and regulations laid down by King Faisal Specialist Hospital and Research Centre Review Board.

### Array hybridization and transcriptome data analysis

Whole genome left ventricular gene expression profiling was performed using Applied Biosystems (ABI) human array ver.2, which contains 31,700 sixty-mer oligonucleotide probes, representing 27,868 individual human genes. Total RNA was extracted and purified from the left ventricular biopsies using Totally RNA Isolation Kit (Applied Biosystems/Thermo Scientitific, Foster City, CA, USA), and quantified with the NanoDrop^®^ ND-1000 Spectrophotometer (Nanodrop Inc., Wilmington, DE, USA). The RNA was further inspected with RNA 6000 Nano Assay kit using 2100 Bioanalyzer (Agilent Technologies, Santa Clara, CA, USA). High quality RNA (1 μg of RNA that has integrity number 8 and above) was used to generate cRNA that was labeled with Digoxigenin-UTPs using Chemiluminescent RT-IVT Labeling Kit v 1.0 (Applied Biosystems). The labeled cRNAs were hybridized onto the arrays for 16 h at 55°C. The arrays were washed and placed into the microarray analyzer for image acquisition. The preliminary data analysis based on image quality was performed on ABI 1700 Chemiluminescent Microarray Analyzer (ABI, Foster City, CA, USA). Array hybridization and data analysis were performed as described previously [15]. Briefly, data normalization and identification of differentially expressed genes was accomplished using Bioconductor packages (Fred Hutchinson Cancer Research Center, Seattle, WA, USA) and Partek Genomics Suite (Partek Inc.). Thereby, genes exhibiting p<0.05, adjusted for multiple comparisons using false discovery rate (FDR) at 5% and the absolute fold changes (FC)>1.5 were considered significantly modulated. The DAVID Bioinformatics Resources [17], Protein ANalysis Through Evolutionary Relationships (PANTHER™) classification systems[18] and Ingenuity Pathways Analysis (IPA) 6.4 (Ingenuity Systems, Mountain View, CA) were used to perform functional annotation and biological term enrichment analysis. The MATLAB software packages (Mathworks, Natick, MA, USA), R and PARTEK Genomics Suite (Partek Inc., St. Lois, MO, USA) were employed for statistical analysis. All microarray data reported here are MIAME compliant and have been submitted to ArrayExpress database (Accession: E-TABM-480).

### Sample preparation for label-free protein in-solution digestion

Seven left ventricular cell lysate samples from the same hearts employed for transcriptome expression were subjected to proteome analysis. Protein concentrations were normalized and equal amounts employed to generate a pool of DCM and CON each. For each group, 100 μg protein was subjected to in-solution tryptic digestion as previously described (30,31). Accordingly, proteins were denatured in 0.1% RapiGest SF at 80°C for 15 minutes, reduced in 10 mM dithiothreitol at 60°C for 30 min, alkylated in 10 mM iodoacetamide (1.0 μL/10 μL) in the dark for 40 min at room temperature and trypsin-digested overnight at 37°C. Samples were diluted with aqueous 0.1% formic acid prior to LC/MS analysis to achieve a load of approximately 2 μg on the analytical column. All samples were spiked with yeast alcohol dehydrogenase (ADH) as internal standard to give 100 *f*mol per injection for absolute quantification.

### Protein identification by LC-MS^E^ system

Label-free quantitative 1-dimensional Nano Acquity liquid chromatography coupled with tandem mass spectrometry on Synapt G2 (Waters, Manchester, UK) used to generate expression data on protein changes between the two groups. The electrospray ionization-mass spectrometry (ESI-MS) analysis and instrument settings were optimized as described previously [19,20]. Briefly, the detector was set up using 2 ng/μL leucine encephalin, and a separate infusion of 500 *f*mol [Glu] 1-fibrinopeptide B was used for mass (m/z) calibration on the Mass Lynx IntelliStart (Waters, Manchester, UK) [19]. All analyses were performed on Triazaic Nano source, and ionization in the positive ion mobility mode nanoESI (Waters, Manchester, UK). Data-independent acquisition (MSE)/ion mobility separation was performed and data acquired over a range of m/z 50–2000 Da using the Mass Lynx programs (ver.4.1, SCN833, Waters, Manchester, UK) as previously described (30, 31).

All samples were analyzed in at least triplicate runs (4–5 times runs repeated on two different occasions). All automated data processing and database search was performed using the Protein Lynx Global Server (PLGS) 2.5 (Waters, Manchester, UK). The generated peptide masses were interrogated against the Uniprot Human specific protein sequence database in parallel with both the PLGS 2.5 and Progenesis QI for protein identification (Waters, UK and Nonlinear Dynamics, Newcastle, UK).

### Proteomic data analysis and Informatics

TransOmics Informatics (Waters Corporation, UK) was used to process and search the data, using a human database containing thousands of reviewed entries from Uniprot as described previously[21]. Normalized label-free quantification was used to plot unsupervised Principal Component Analysis (PCA) against significantly regulated proteins only (ANOVA; p ≤ 0.05) with ≥3 peptides ID and a fold change >1.5, using ADH as an internal standard with Progenesis QI for proteomics (Nonlinear Dynamics, Newcastle, UK).

### Correlation analysis for differentially expressed entities

The differentially expressed genes (DEGs) and proteins (DEPs) were mapped to their respective NCBI Gene Symbols using UniProt Knowledgebase (UniProtKB) and IPA Knowledgebase, using two different approaches to search for concomitant changes in both transcriptomic and proteomic datasets at functional/pathway levels: (1) the Venn diagram approach was used for finding overlapping genes/proteins between transcriptomic and proteomic datasets (2) subjecting DEGs, DEPs and the overlapping genes/proteins to several functional annotation, molecular pathways, and network analyses by several bioinformatics tools, such as (a) DAVID gene ontology (GO) enrichment analysis, (b) IPA functional and pathway analyses, (c) upstream regulator analysis and (d) PANTHER™ classification systems for biological process and pathway analyses, and ReactomeFI[22], regarding p< 0.05 as statistically significant.

### Validation of the transcriptome and proteome data

As a validation of our transcriptome and proteome results, we used four different datasets acquired on independent human cardiac samples with heart failure using microarray and next generation sequencing technologies. First dataset was from GSE3585 consisting 12 independent heart biopsies, generated using Affymetrix HG-U133A array in left ventricles of 7 DCM and 5 controls were analyzed[16]. Two were excluded from further analysis due to poor QC. Second validation dataset was from GSE 57345 which included 218 samples (82 DCM and 136 normal hearts) from Liu et al [23] that was generated using Affymetrix Human Exon ST1.1 arrays. The raw microarray data were normalized by the GC Robust Multi-array Average (GC-RMA) algorithm [24,25]. The third validation dataset was a transcriptome profiling using next-generation sequencing data form Yang et al [26] (GSE46224), which consisted of RNA-Seq data for 40 human cardiac samples for NF (non-failing normal control), ischemic cardiomyopathy (ICM), non-ischemic cardiomyopathy (NICM), ICM with left ventricular assisted device (ICM+LVAD), and NICM+LVAD. However, we used only data from NICM (n = 8, pre LVAD) and normal controls (n = 8) in our validation. The normalized data that the authors provided has been used for subsequent analyses. Finally, the last dataset was from Liu et al [23] (GSE 46224) for RNA-Seq data from two DCM and three normal individuals. Unpaired t-tests were performed to determine significant changes in gene expression between DCM vs normal and Multi Experiment Viewer (MeV4.0)[27] was used to perform two-dimensional hierarchical clustering by Pearson correlation with average linkage clustering.

## Results

### Global transcriptome and proteome profiling in dilated cardiomyopathy

**Fig 1:**
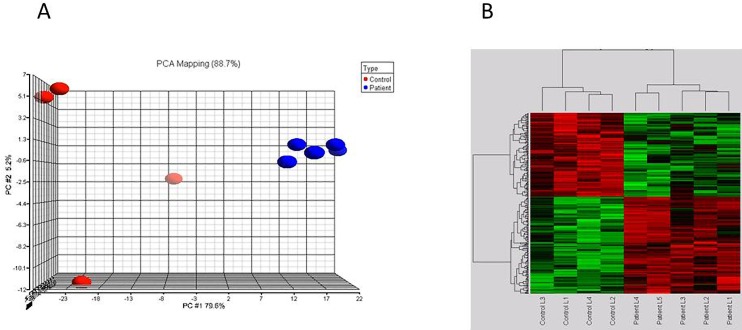
(A) **Unsupervised principal component analysis (PCA) clearly distinguishing DCM patients from normal controls. The blue spheres refer to DCM patients, and red normal controls**. (B) **Heatmap of genes that were significantly modulated in DCM. Hierarchical clustering distinguished individuals as either DCM or normal controls.** Red and green denote highly and weakly expressed genes, respectively.

We analyzed the whole-genome mRNA expression using ABI 1700 microarray analyzer ver.2 (Applied Biosystems, Foster City, CA, USA). Overall, 18,983 out of the 31,700 sixty-mer oligonucleotide probes were expressed, based on the manufacturer’s criteria of signal to noise ratio of >3, indicating a 99.9% confidence that the signal is above the background. Furthermore, 1549 probes corresponding to 1211 genes were differentially expressed. Of these, 597 were downregulated and 614 upregulated, showing variation of ≥1.5-fold at FDR<5% (S1 Table). The DCM patients were clearly distinguishable from normal controls by an unsupervised PCA showing 90% of the variance in the data matrix and two-dimensional hierarchical clustering of samples and genes (Fig 1A and 1B, respectively).

### Global protein expression in human left ventricles

We used a label-free MS-based tool for quantitative and comparative expression analysis of the protein changes in disease. Approximately 1786 proteins were identified, representing >500 unique species. Some 269 proteins were differentially expressed between the two groups (*p<*0.05, >1.5-fold change, and power ≥ 0.9 at FDR~3%) (S2 Table). The majority of the proteins were upregulated (S2 Table) in concordance with the observations using the PCA plot generated from two-dimensional gel electrophoresis dataset (data not shown). Among the most significantly upregulated proteins were GRK5, APOA2, IGHG3, ANXA6, 90 kD cytosolic heat shock protein (HSP90AA1), and ATP5C1 (p<0.01), while the most significantly downregulated proteins included GSTM5, cytochrome C oxidase copper chaperone (COX17), caveolin 1 (CAV1) and annexin A3 (ANXA3). The 269 proteins could be clustered into two distinct groups of cases and controls using the PCA analysis (S1 Fig).

### Concomitant differential proteomic and transcriptome expressions

**Fig 2:**
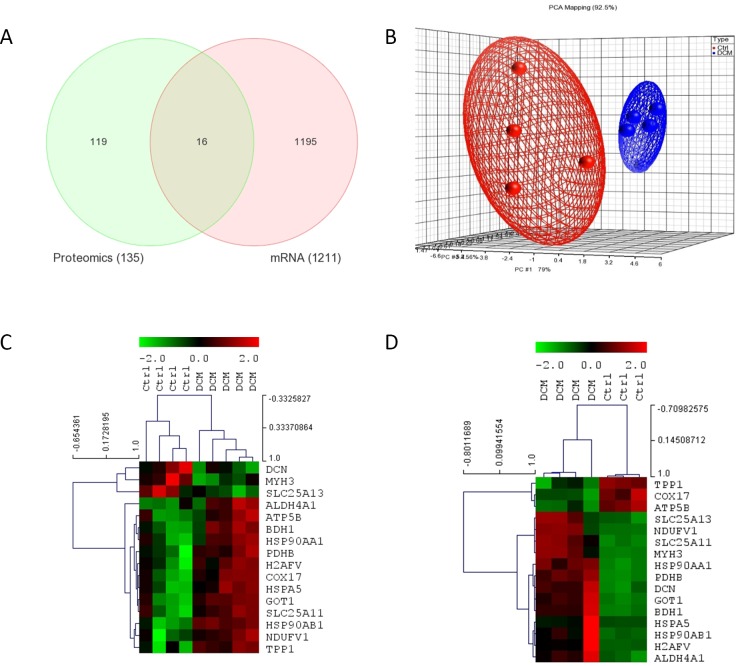
(A) **Venn diagram representing the overlapping between the differentially expressed proteins and genes. Sixteen proteins/genes were commonly identified in both global gene and protein profiling.** (B) Unsupervised principal component analysis (PCA) of the commonly dysregulated proteins/genes separating samples into DCM patients and normal controls. The blue spheres refer to DCM patients and red normal controls. (C-D) Unsupervised two-dimensional hierarchical clustering of proteomic and transcriptomic datasets using commonly dysregulated proteins/genes (C and D, respectively). The hierarchical clustering revealed two main clusters, one composed of DCM patients and another composed of normal controls. Highly expressed genes are indicated in red, intermediate in black, and weakly expressed in green.

The DEGs and DEPs were mapped to their respective NCBI Gene Symbols using UniProt Knowledgebase (UniProtKB) and IPA Knowledgebase and overlapping genes/proteins between transcriptomic and proteomic datasets were found using the Venn diagram approach. At least sixteen entities were commonly up/downregulated in both protein and RNA expression platforms, ten of which displayed concordant expression patterns (Fig 2A, Table 1). The concomitantly upregulated entities included soluble glutamic-oxaloacetic transaminase 1 (GOT1), ALDH4A1, PDHB, type 1 3-hydroxybutyrate dehydrogenase (BDH1), Ca^2+^-binding mitochondrial carrier protein subtypes (SLC2A11), HSP90AA1, HSP90AB1, H2AFV, HSPA5 and 51 kDa NADH dehydrogenase (ubiquinone) flavoprotein 1 (NDUFV1). Apart from these uniformly charging entities, three others, decorin (DCN), MYH3 and SLC25A13 exhibited a decrease in mRNA expression in contrast to increased protein expression, while the ATP5B, COX17 and TPP1 displayed the opposite trends.

**Table 1:** The 16 overlapping differential expressed genes/proteins in transcriptomic and proteomic data in the study.

	mRNA			Proteome		
Symbol	Probe ID	FC	P-value	UniprotID	FC	P-value
ALDH4A1*	224882	1.518	0.034	P30038	1.579	0.011
ATP5B	155269	4.019	0.046	P00830	-1.642	0.002
BDH1	122515	3.260	0.008	Q02337	1.922	0.002
COX17*	161007	3.325	0.019	Q14061	-3.245	0.019
DCN	707818	-2.028	0.037	P07585	1.716	0.002
GOT1	211439	5.833	0.025	P00504	2.198	0.001
H2AFV	203036	2.957	0.036	P08985	3.267	0.012
HSP90AA1*	160490	5.010	0.040	Q90474	2.198	0.000
HSP90AB1*	197185	2.472	0.008	P41887	3.351	0.009
HSPA5	129530	3.820	0.023	Q9LKR3	4.010	0.035
MYH3	116176	-2.067	0.021	P13541	2.072	0.002
NDUFV1	183186	1.994	0.038	P25708	3.120	0.011
PDHB	171509	1.922	0.027	P11966	2.237	0.000
SLC25A11	110914	9.392	0.045	P22292	2.113	0.001
SLC25A13*	121335	-2.053	0.031	Q9QXX4	3.263	0.021
TPP1	158867	4.918	0.038	O14773	-1.563	0.009

Accession numbers are the protein accession numbers; FC, fold change

*Genes in asterisks are also significantly dysregulated in independent RNA-Seq validation dataset from Yang et al [26].

An unsupervised PCA of the 16 genes clearly distinguished individuals as DCM and normal controls (Fig 2B). We then employed two-dimensional hierarchical clustering of samples which revealed clear gene deregulation patterns defining two main transcriptome and proteome clusters, one composed of cases, and the other normal controls (Fig 2C and 2D, respectively).

### Gene ontology enrichment, pathway and network analyses

**Fig 3:**
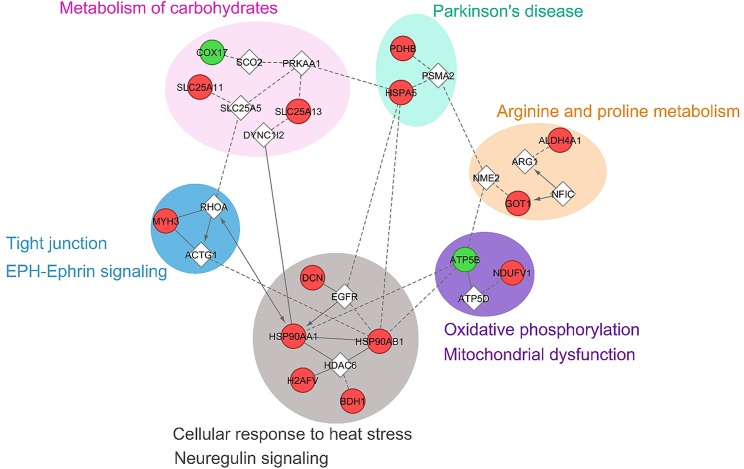
Functional interaction network of 16 genes. Genes were clustered according to their associated pathways, which are shaded with a different color. Green nodes indicate down-regulated, red, up-regulated, and linker genes (non-colored nodes). The edges represent interactions between genes, with arrows indicating directed interactions and dotted lines indicating predicted relationships.

**Fig 4:**
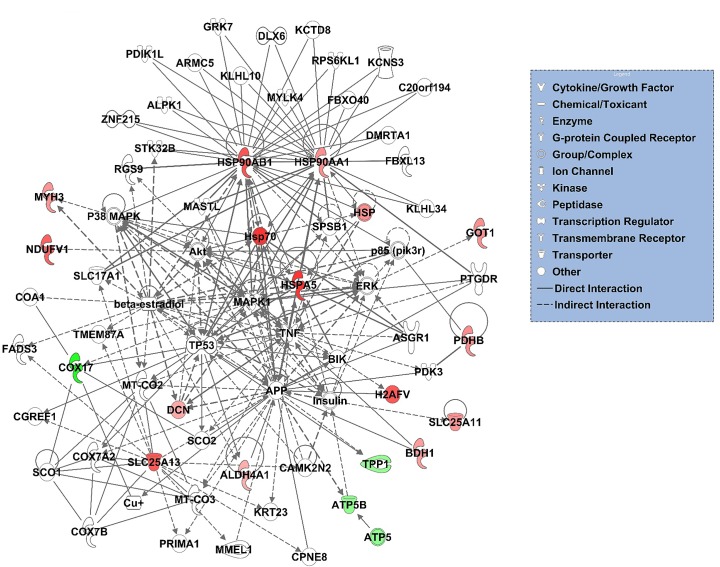
Gene interaction network analyses of 16 commonly dysregulated proteins/genes based on the Ingenuity knowledge base. Green indicates down-regulated, and red, up-regulated. The color intensity is correlated with fold change. Straight and dashed lines represent direct or indirect gene to gene interactions, respectively.

Projecting the 16 overlapping entities onto the protein functional interaction networks using the ReactomeFI revealed network patterns highly enriched in carbohydrate metabolism, Parkinson’s disease, arginine and proline metabolism, oxidative phosphorylation/mitochondrial dysfunction, cellular response to heat stress/neuregulin signalling and tight junction/EPH-Ephrin signaling pathways (Fig 3). The Ingenuity knowledge base analysis for the 16 genes revealed top networks related to, among others, organismal injury and abnormalities, post-translational modification, tissue morphology, lipid metabolism, cardiovascular system development/function (Fig 4). GO enrichment analysis associated with the 16 genes were also identified. The most significantly overrepresented GO categories were related to oxidation-reduction catalytic activity, metabolic processes, glycolysis and energy pathways, which were consistent with the categories identified by PANTHER analysis.

### Concomitant differential expression data validation

**Fig 5:**
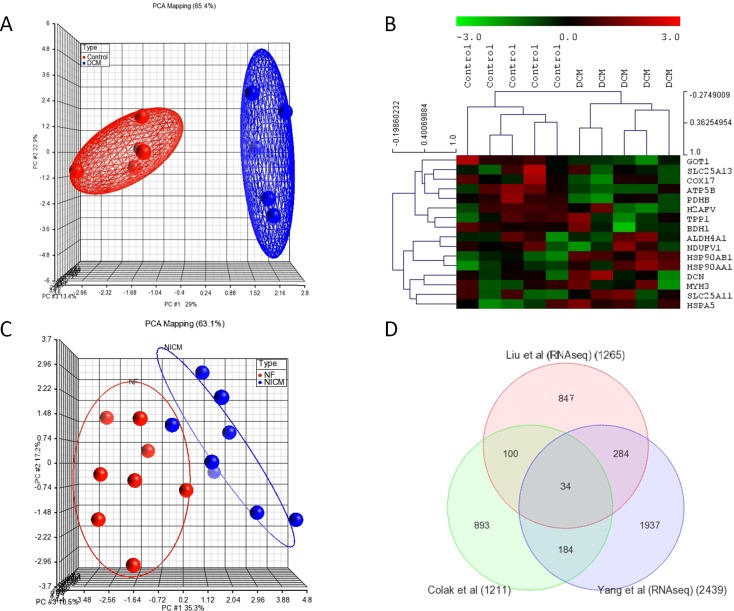
Validation analyses using independently performed microarray and RNAseq datasets. (A) **The PCA and (B) unsupervised hierarchical clustering using our 16 gene set discriminated individuals as DCM and controls in Barth et al.’s [16] microarray data**. Samples are in the columns and genes are in the rows (gene symbols are listed on the right). The expression level of each gene across samples is scaled to [−3, 3] interval. The expression levels are depicted using a color scale as shown at the top of the figure. (C) **PCA analysis using RNA-Seq dataset for (non-ischemic cardiomyopathy (NICM) (n = 8) and normal controls (n = 8) from Yang et al [26].** (D) **Venn diagram representing the genes common to DEGs in our DCM patients (Colak et al[15]) with DEGs in validation datasets from datasets from Yang et al [26] and Liu et al [23] for RNA-Seq data for independent samples from human failing heart**.

To validate our results, we analyzed four independently acquired transcriptomic datasets generated by microarray and next generation sequencing (RNA-Seq) platforms for independent human cardiac samples with heart failure, as detailed in the Materials and Methods section. The first validation dataset was from Barth et al.’s [16] microarray data which consisted of ten left ventricular heart biopsies (five DCM and five controls) generated on the Affymetrix Array U133A array [16], in which the PCA and unsupervised hierarchical clustering using our sixteen genes/proteins separated the individuals as either patients or controls (Fig 5A and 5B, respectively). Similarly, our gene signature was sufficient to cluster the samples in Yang et al [26]’s RNA-Seq data as non-ischemic cardiomyopathy (NICM) (n = 8) and normal controls (n = 8) (Fig 5C). Furthermore, the unsupervised hierarchical clustering of samples from Liu et al [23], which included 82 DCM and 136 normal hearts, resulted in two main transcriptome clusters, one was mainly from normal hearts and the other one from DCM patients (S2 Fig).

As a further validation, we compared the DEGs in independent patients with failing human heart by interrogating the independent datasets including the RNA-Seq datasets from Liu et al [23] and Yang et al [26] with DEGs in our patients, which revealed significant overlaps (P-value < 0.01) (Fig 5D and S1 Table).

### Functional and pathway overlap between proteomic and transcriptome expressions

**Fig 6:**
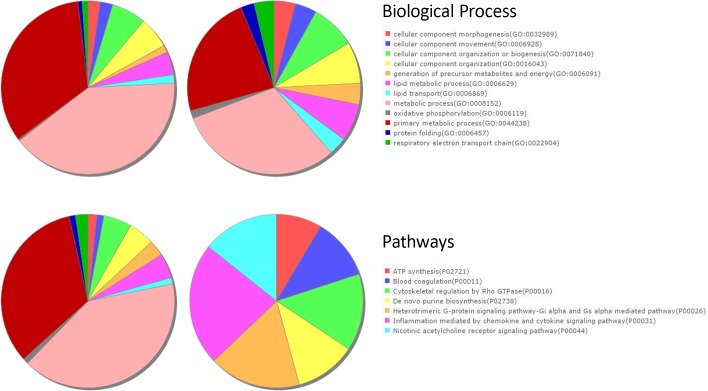
GO Biological Process and pathway analyses of differentially expressed genes (DEGs) and proteins (DEPs) using the PANTHER classification system. (A-B) Pie charts displaying significantly enriched biological processes respectively, and (C-D) signaling pathways associated with DEGs and DEPs.

To test for concomitant functional changes and pathway alterations in the transcriptome and proteomic datasets, we subjected the DEGs and DEPs to functional annotation, molecular pathways, and network analyses using various analytical tools. Based on the IPA analysis, DEGs were significantly enriched with, among others, cardiovascular and nervous system development and function, while significantly altered canonical pathways included oxidative phosphorylation, mitochondrial dysfunction and TCA cycle. We further identified altered biological processes, molecular functions and pathways using PANTHER^TM^ classification system[18], exhibiting significantly altered genes and pathways related to various cardiac related functions (Fig 6A and 6C).

The DEPs included a diverse spectrum of proteins, whose functions have not been fully elucidated yet. By far the largest number of the altered entities belonged to enzyme families, and classical signaling entities included those involved guanine nucleotide protein-coupled receptor (GPCR) signaling and various other cardiovascular-related receptor signaling and protein kinases (S2 Table, S3 Fig). The identified proteins were mapped to multiple networks including those implicated in cardiovascular disorders/system development, abnormal morphology, smooth muscle contraction, ion homeostasis and nitric oxide signaling pathway in IPA knowledgebase (S4 Fig). The top associated network functions included developmental hereditary and metabolic disorders, altered canonical pathways included the LXR/RXR action (p = 7.9E-10), FXR/RXR activation (p = 1.2E-09), acute phase response signaling (p = 2.0E-08), upstream regulators included PLN (p = 4.9E-12), APP (p = 1.45E-08) and microtubule-associated protein Tau (MAPT) (p = 2.5E-09), while molecular and cellular functions included several systems involving 26 molecules in organ morphology, 23 skeletal/muscle and 29 cardiovascular system developments. Changes in cardiovascular proteins included those linked to abnormal cardiomyocyte morphology, endothelial cell movement and coupling, cardiac muscle contractility and conduction, basilar artery and left ventricular diameters, blood vessel and vascular tissue permeability (S4 Fig).

**Fig 7:**
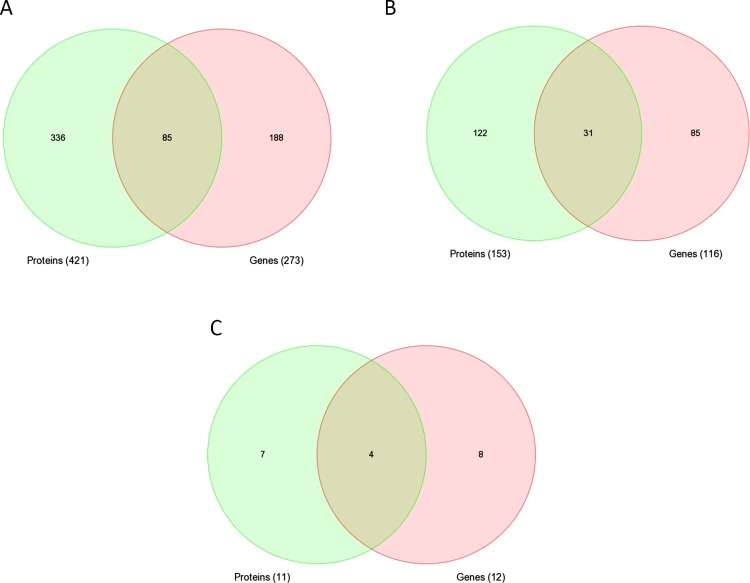
Venn diagrams representing overlap of (A) predicted upstream regulators, (B) enriched GO biological processes, and (C) KEGG pathways between differentially expressed genes and proteins.

The GO enrichment analyses for both DEGs and DEPs using PANTHER^TM^ classification system displayed diverse overlapping functional categories and pathways, including metabolic processes, oxidative phosphorylation and cellular component morphogenesis (Fig 6). Furthermore, DAVID and IPA analyses of DEGs and DEPs revealed 85 common upstream regulators, whereby both lists were enriched with 31 GO biological processes and 4 Kegg pathways (Table 2; Fig 7; S3 and S4 Tables).

**Table 2:** Overlapping KEGG pathways for mRNA and proteomics in in dilated cardiomyopathy.

Term	mRNA		Proteome	
	P-value	Gene	P-value	Proteins
**hsa04260 (Cardiac musclecontraction)**	8.03E-04	ATP1B1, COX7A2, UQCRC1, CYC1, ATP1B4, CACNB2, COX4I1, COX5A, UQCRFS1, UQCRQ, COX5B, TPM4, UQCR10, COX6B1, COX6A1	0.0025	UQCRC2, ATP2A2, MYH7, ATP1A1, ATP1A2, TPM1, TPM3
**hsa05010 (Alzheimer's disease)**	5.30E-06	NDUFB5, COX7A2, UQCRC1, NDUFA8, NDUFB8, ATP5B, CYC1, NDUFAB1, COX4I1, NDUFA10, UQCRFS1, COX5A, UQCRQ, COX5B, NDUFB2, SDHA, NDUFS6, UQCR10, MAPT, NDUFV1, ATP2A1, COX6B1, COX6A1, ATP5O, ATP5A1, FAS, NDUFS3, ATP5H, NDUFS5	0.002	UQCRC2, BID, HSD17B10, NDUFA2, ATP2A2, CALML3, ATP5B, NDUFV1, CYCS, ATP5C1
**hsa05012 (Parkinson's disease)**	6.76E-09	NDUFB5, UQCRC1, POLR2L, NDUFB8, ATP5B, CYC1, DNAH3, NDUFAB1, UQCRFS1, COX5A, UQCRQ, COX5B, NDUFB2, NDUFS6, AP2B1, UQCR10, COX6B1, ATP5O, NDUFS3, ATP5H, NDUFS5, COX7A2, NDUFA8, RCOR1, COX4I1, NDUFA10, SOD1, PPARGC1A, SDHA, HDAC2, NDUFV1, COX6A1, ATP5A1	0.008	UQCRC2, NDUFA2, SLC25A5, ATP5B, NDUFV1, CYCS, ATP5C1, VDAC2
**hsa05016 (Huntington's disease)**	4.93E-07	NDUFB5, UQCRC1, POLR2L, NDUFB8, ATP5B, CYC1, DNAH3, NDUFAB1, UQCRFS1, COX5A, UQCRQ, COX5B, NDUFB2, NDUFS6, AP2B1, UQCR10, COX6B1, ATP5O, NDUFS3, ATP5H, NDUFS5, COX7A2, NDUFA8, RCOR1, COX4I1, NDUFA10, SOD1, PPARGC1A, SDHA, HDAC2, NDUFV1, COX6A1, ATP5A1	0.015	UQCRC2, NDUFA2, SLC25A5, ATP5B, NDUFV1, CYCS, ATP5C1, CLTC, VDAC2

## Discussion

The present investigation characterized alterations in left ventricular gene and protein expression in DCM, in order to decipher disease-specific or -related biomarker signatures that could enhance the diagnosis and prognosis of the disease. Several mRNA and protein species were differentially expressed in DCM compared to non-failing left ventricular heart tissues. A large number of the upregulated entities and pathways belonged to families involved in cardiomyocyte contractility, cell survival, cell cycle and energy metabolic patatin-like phospholipase domain containing 3 (PNPLA3), processes, as indicated by the abundance of altered ATP, ATPases and mitochondrial enzyme activities. Functionally, by far the greatest majority of these entities belong to a spectrum of enzyme families mediating a variety of activities, including DNA/RNA regulation/processing, cellular metabolism, cell cycle control and homeostasis, whose cardiac-related functions remain largely obscure. Hence, our data furnishes compelling evidence for the marked changes in metabolic and stress response processes in DCM that still need to be deciphered.

Of particular interest was the observation that only a few of the most significantly up/downregulated proteins such as the superoxide dismutase (SOD2), HSP90AA1 or GOT1, could be allotted a cardiac-related function, or may have been implicated in cardiovascular disease thus far. Among these, the SOD2, which is thought to play a role in resisting external superoxide stress, acid tolerance and acid-adaptive response, has been associated with various diseases, including DCM, sporadic motor neuron disease and cancer[28–32], while an increase in the HSP90AA1 activity may represent a response to ischemia and reperfusion injury[33], for example. The GOT1 acts as a scavenger of glutamate in brain neuroprotection and has been implicated in the pathogenesis of isoproterenol-induced myocardial injury and increased myocardial antioxidant capacity in rats [34].

On the other hand, many of the differentially expressed proteins have not been linked to DCM yet, but rather implicated in diverse processes whose relationship to cardiovascular function remains to be elucidated. These include the HSP90AB1, UDP-N-acetylenolpyruvoyl glucosamine reductase (MURB), EFTU), neutral alpha-glucosidase C (GANC), VG03, early response growth protein and leucine-rich repeat serine/threonine protein kinase 1 (LRRK1), all of which showed a 10-fold change in DCM. Thus, for example, the FAD cofactor MURB is involved in cell wall formation and biogenesis as well as peptidyl glycan biosynthesis pathways, EFTU is thought to be involved in mitochondrial translation and translational elongation, organelle organization, and BDH1 believed to be involved in cellular ketone body metabolic biosynthesis, catabolic processes as well as cellular lipid and small molecule metabolism. However, currently there is lack of information regarding their potential role in cardiovascular function. Besides, several other significantly altered proteins (>5-fold) including ankyrin repeat domain-containing protein 2 (ANKR2), Ig kappa chain V-I region Rei (KV115), hemoglobin beta chain (HBB), DENN domain-containing protein 5A (DEN5A), patatin-like phospholipase domain containing 3 (*PNPLA3)*, tRNA-dihydrouridine synthase [NAD(P)(^+^)]-like isoform 3 (DUS3L), appear not only to be even further remotely linked to classical cardiovascular functionality, but also to await full characterization in humans. Having said that, their down-/upregulation indeed points to their potential involvement in cardiomyocyte modification(s) as an integral component of events occurring in DCM. Therefore, in-depth studies are necessary to ascertain the actual role of these diverse, yet to be deciphered transcriptome and proteome changes in DCM pathways. Understandably, several modest changes in many other proteins were also observed commensurate with classically expected changes in DCM and heart failure. Thus, although these alterations may occur independently of the underlying disease etiology, it is also known that the greater part of myocardial changes is triggered by chronic neurohumoral activation and abnormal mechanical load[35]. Hence, these manifestations may constitute components of the biological processes involving these changes, which probably form part of a vicious circle that enhances the progression of heart failure, a subject that continues to attract focused research attention.

Interestingly, at least ten of the entities displaying significant differential expression between disease and controls exhibited similar trends in their expression on the two platforms. One of these, the SLC25A11, plays an important role in several metabolic processes and appears to be downregulated in ischemic stroke[36]. Another entity displaying similar tendencies, the HSPA5, probably plays a role in facilitating the assembly of multimeric protein complexes inside the endoplasmic reticulum, and may be required for the correct protein folding and misfolded protein degradation. However, no relationship to disease has been established yet for these interactions. The NDUFV1 constitutes a core subunit of the mitochondrial membrane respiratory chain NADH dehydrogenase (Complex I) belonging to the minimal assembly required for catalysis in the transfer of electrons from NADH to the respiratory chain, and has recently been implicated in DCM[37] as well as remodeling in ischemic heart failure[38]. PDHB forms part of a complex that catalyzes the overall conversion of pyruvate to acetyl-CoA and CO_2_, thereby linking the glycolytic pathway to the tricarboxylic cycle. Such concomitant alterations in transcriptome and proteome expression constitutes a potential platform for defining markers for DCM, calling for further efforts to be exploited in this direction. Furthermore, the COX17 has been implicated in various processes including brain and heart development, generation of precursor metabolites and energy, and its deficiency has been associated with heterogeneous clinical phenotypes[39]. DCN is a putative currently uncharacterized protein that has been linked to skeletal muscle tissue development, response to aging, kidney development and wound healing. It is thought to be involved in cardiac remodelling[40,41] and to contribute to attenuating the progression of atherosclerosis [42]. The SLC25A13 catalyzes the Ca^2+^-dependent exchange of cytoplasmic glutamate with mitochondrial aspartate across the mitochondrial inner membrane, and may have a function in the urea cycle, while TPP1 is a lysosomal serine protease whose mRNA expression is increased during exercise and training and linked to ageing process[43]. Mutations in the gene have been associated with various diseases.

Thus, our study demonstrated that multiple interlinked processes are indeed involved in the pathways to DCM and exudes the power of transcriptome expression and label-free expression proteomics towards biomarker discovery for DCM. Accordingly, it is anticipated that combinations of different analysis platforms, rather than use of a single method, would render themselves more reliable to answer complex biological questions. However, while great enhancement of our understanding of differential gene and protein expression has been made possible through the advent of such microarray-based and similar techniques[16,44–46], it appears nonetheless that much more still needs to be leant about these techniques. Thus, apart from possible technically related uncertainties in sample preparation and reliability of the whole-genome analytical techniques especially for those entities displaying low expression levels, such differences may also be influenced by the signaling networks linked to their expression. In fact, the quality control test indicated that some of such changes eventually showed similar trends, suggesting in deed that these dissimilarities might have been due to technical inconsistencies. Besides, a validation analysis with other data sets using a different microarray platform demonstrated great concordance of our results. Notably also, GO analyses of the DEGs and DEPs revealed significant overlap with enrichment of genes/proteins related to many processes including not only those that are likely to be altered in the progression of DCM to overt heart failure, but are also linked to other disease such as Alzheimer’s disease (AD) for example, which strengthens the notion that these two diseases might share some common pathway that need to be deciphered[15]. Therefore, our approach produced comparatively reliable and informative results with the array platforms employed, especially if considered together with similar gene expression by different platforms[47–49]. Hence, clinically useful inferences continue to be derived from such studies.

In summary, the concomitant employment of transcriptome and proteome expression to evaluate alterations in DCM in the present study led to the identification of sixteen commonly altered entities, providing further insight into the mechanism of heart muscle disease pathways. It also led to the identification of novel entities and pathways whose cardiac functions have yet to be deciphered. Together with an integration of clinical and biochemical information, this data should contribute towards better management of the disease. Importantly also, the resemblance of DCM with disorders, such as cancer or neurodegenerative disorders, in the pattern of differential expression of several proteins, molecular functions and pathways also reveals a potential link of these diseases at the level of energy metabolism and maintenance of cellular structural integrity.

## Supporting Information

S1 Fig Principal Component Analysis using the 268 identified proteins with significant difference in expression (combined P< 0.05, 1.5-Fold change and Power 0.9) between left ventricles of DCM and control samples. Their expression changes allow for clear separation into two distinct sample groups. The numbers of the identified proteins are indicated in the grey colour, while purple = DCM, and blue = Control. The PCA plots were generated using the Progenesis LC-MS (Nonlinear Dynamics, UK).
(DOCX)

S2 Fig Unsupervised hierarchical clustering of samples from Liu et al [23], which included 82 DCM and 136 normal hearts. Samples are in the columns and genes are in the rows (gene symbols are listed on the left). The expression level of each gene across samples is scaled to [−4, 4] interval. The expression levels are depicted using a color scale as shown at the bottom of the figure.
(DOCX)

S3 Fig Graphical representation of the list in S1 Table of most significantly altered enzyme families in dilated cardiomyopathy. (DOCX)

S4 Fig The functional interaction network with molecule activity predictions for differentially expressed proteins that are related to cardiovascular development system. Green indicates down-regulated, and red up-regulated. Blue/orange line indicates predicted inhibition/activation of a gene.
(DOCX)

S1 Table List of the differentially expressed genes, showing 597 downregulated and 614 upregulated entities, at ≥1.5-fold variation level. (XLS)

S2 Table List of some 269 differentially expressed proteins (*p<*0.05, >1.5-fold change, and power 0.9 at FDR~3%). The majority of the proteins were upregulated in concordance with the observations using the PCA plot generated from two-dimensional gel electrophoresis dataset.
(XLS)

S3 Table List of the proteomic-target molecules in the data set showing common upstream regulators for the altered proteins based on DAVID and IPA analyses, enriched with 31 GO biological processes and 4 Kegg pathways. (XLS)

S4 Table List of the proteomic-target molecules in the data set showing common biological processes for the altered proteins based on DAVID and IPA analyses, enriched with 31 GO biological processes and 4 Kegg pathways. (XLS)
