# Supplementary figures and images for: Integrated Left Ventricular Global Transcriptome and Proteome Profiling in Human End-Stage Dilated Cardiomyopathy
Source: PLoS One. 2016 Oct 6;11(10):e0162669. doi: 10.1371/journal.pone.0162669 (PMC5053516; doi:10.1371/journal.pone.0162669)

**S1 Fig**

**
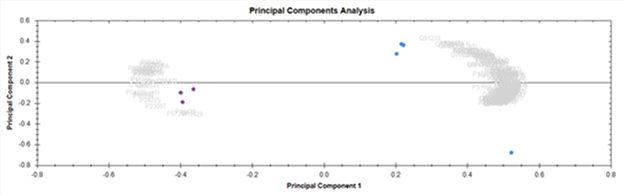
**

Supplement: S1 Fig — Their expression changes allow for clear separation into two distinct sample groups. The numbers of the identified proteins are indicated in the grey colour, while purple = DCM, and blue = Control. The PCA plots were generated using the Progenesis LC-MS (Nonlinear Dynamics, UK). (DOCX) [file pone.0162669.s001.docx]

**S4 Fig**


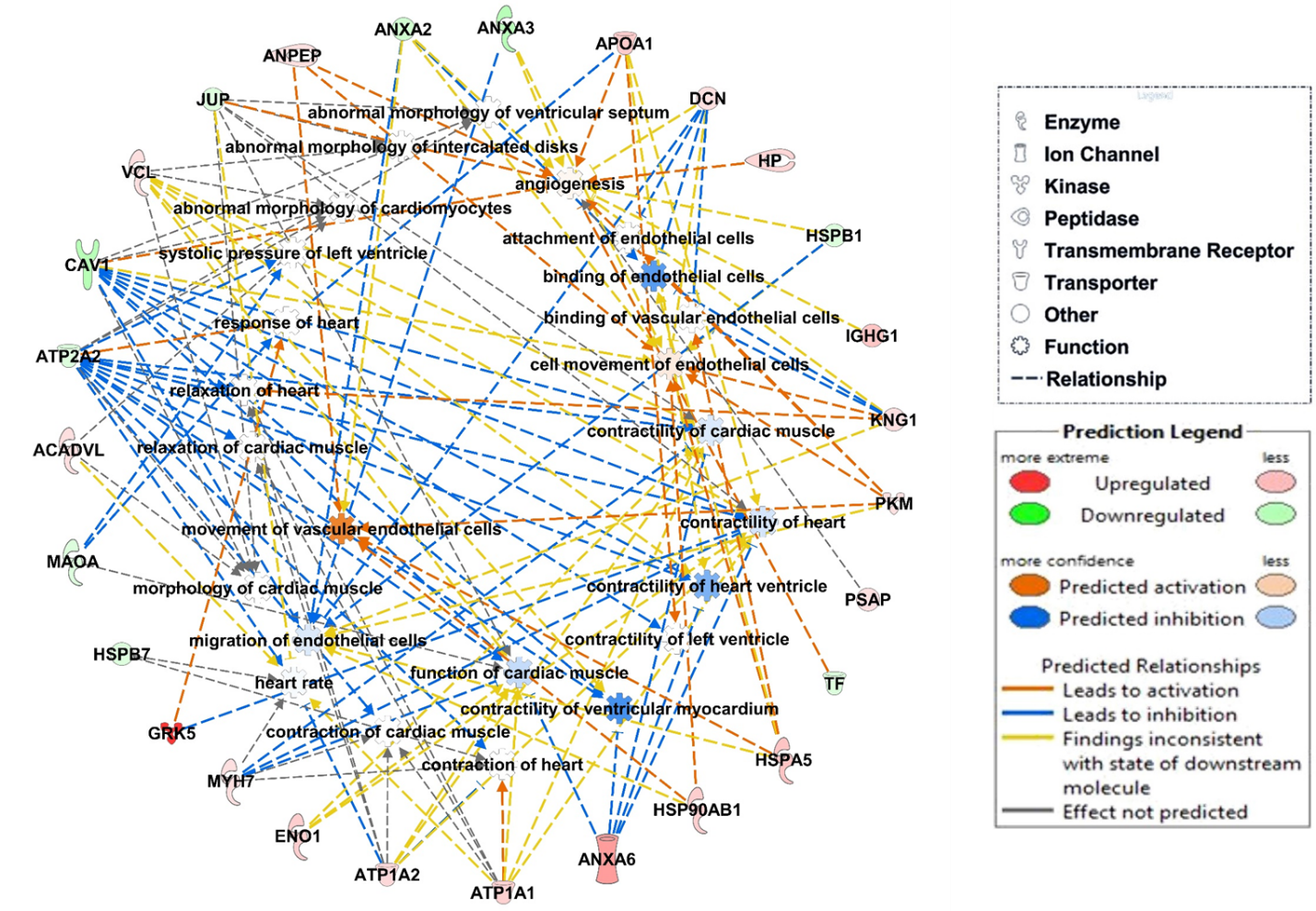

Supplement: S4 Fig — Green indicates down-regulated, and red up-regulated. Blue/orange line indicates predicted inhibition/activation of a gene. (DOCX) [file pone.0162669.s004.docx]
