# Supplementary material for: Integrated Left Ventricular Global Transcriptome and Proteome Profiling in Human End-Stage Dilated Cardiomyopathy
Source: PLoS One. 2016 Oct 6;11(10):e0162669. doi: 10.1371/journal.pone.0162669 (PMC5053516; doi:10.1371/journal.pone.0162669)
Supplement: S2 Fig — Samples are in the columns and genes are in the rows (gene symbols are listed on the left). The expression level of each gene across samples is scaled to [−4, 4] interval. The expression levels are depicted using a color scale as shown at the bottom of the figure. (DOCX) [file pone.0162669.s002.docx]

**S2 Fig**


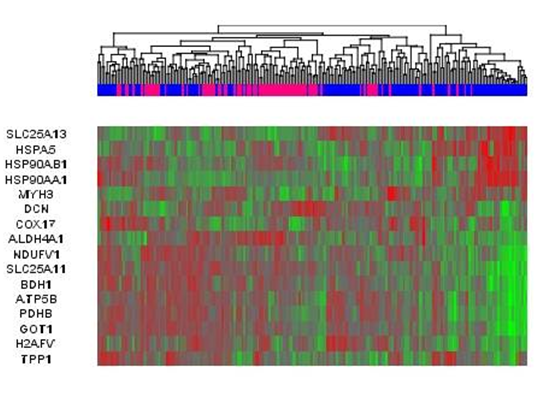


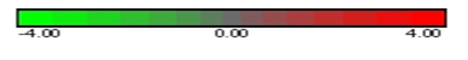


Unsupervised hierarchical clustering of samples from Liu et al ([23](#_ENREF_23)), which included 82 DCM and 136 normal hearts. Samples are in the columns and genes are in the rows (gene symbols are listed on the right). The expression level of each gene across samples is scaled to [−4, 4] interval. The expression levels are depicted using a color scale as shown at the bottom of the figure.
